# The effect of 4.3 GHz high-power microwave exposure on human corneal epithelial cells

**DOI:** 10.3389/fcell.2026.1729198

**Published:** 2026-01-22

**Authors:** Anning Gao, Xuelong Zhao, Shuang Wu, Xiaoman Liu, Xinyu Wang, Luhao Tan, Zhihui Li, Guofu Dong, Changzhen Wang

**Affiliations:** 1 Basic Medical College of Anhui Medical University, Hefei, China; 2 Beijing Key Laboratory for Radiobiology, Beijing Institute of Radiation Medicine, Beijing, China

**Keywords:** apoptosis, corneal epithelial cells, high-power microwave, mitochondrial dysfunction, MTOR signaling, non-thermal effects, oxidative stress

## Abstract

**Introduction:**

High-power microwave (HPM) exposure can produce biological effects in cells, but the specific characteristics and mechanisms of these effects in ocular tissues remain poorly defined. This study aimed to investigate the biological responses of human corneal epithelial cells (HCE-T) to 4.3 GHz HPM exposure, with a focus on moderate-dose effects.

**Methods:**

HCE-T cells were exposed to 4.3 GHz HPM at average specific absorption rates (SARs) of 1.64, 3.28, and 8.2 W/kg. Cellular responses were evaluated by measuring cell viability, reactive oxygen species (ROS) generation, mitochondrial membrane potential, and apoptosis at multiple time points. Transcriptomic analysis was performed to identify underlying molecular pathways.

**Results:**

Moderate-dose exposure (3.28 W/kg) resulted in the most pronounced cellular effects, including early and significant ROS elevation, marked collapse of mitochondrial membrane potential, the highest apoptosis rate, and sustained inhibition of proliferation. Transcriptomic profiling showed strong suppression of the mTOR signaling pathway, upregulation of TSC2, and activation of Polycomb-mediated chromatin remodeling, suggestive of autophagy induction and irreversible cell cycle arrest. In contrast, low-dose exposure (1.64 W/kg) primarily activated DNA repair and adaptive pathways, while high-dose exposure (8.2 W/kg) predominantly disrupted metabolic and membrane signaling with a trend toward recovery.

**Discussion:**

These findings demonstrate that moderate-dose 4.3 GHz HPM exposure induces a uniquely strong stress response in HCE-T cells, characterized by oxidative stress, mitochondrial dysfunction, and activation of stress-related signaling pathways. These results highlight the importance of considering specific exposure conditions in assessing HPM bioeffects and ocular safety.

## 1 Introduction

High-power microwaves (HPM) generally refer to strong electromagnetic radiation with operating frequencies between 0.3 and 300 GHz (wavelengths of 1 mm to 1 m) and peak powers exceeding 100 MW (Mumtaz et al., 2024; Liu et al., 2014). Pulsed HPM technology has been under development for several decades (Mumtaz et al., 2024), with notable innovations in high-frequency nanosecond pulsed HPM (Rana et al., 2024). HPM has attracted attention in biomedical research due to its potential to influence multiple biological systems (Mumtaz et al., 2023).

Current studies have shown that nanosecond pulsed HPM (pulse width 1–300 ns) can establish transient ultrahigh-frequency electric fields (up to 27 kV/cm) in biological tissues in the gigahertz range. The underlying mechanism involves dynamic polarization of the membrane bilayer, which may induce GHz-level electroporation (Rana et al., 2024). This transient opening of nanoscale pores, lasting from microseconds to milliseconds, significantly enhances the transmembrane delivery of therapeutic macromolecules such as nucleic acid drugs and monoclonal antibodies, with an efficiency 3–5 orders of magnitude higher than that of conventional electroporation techniques. Importantly, even microwave exposure alone may cause both thermal injury and non-thermal effects in biological tissues (Yoon et al., 2020; Zhang et al., 2020; Shaw et al., 2021). These mechanisms involve disturbances of membrane potential, conformational changes in proteins (Mumtaz et al., 2022), mitochondrial structural and functional damage at multiple levels (such as swelling, cristae disruption, membrane potential loss, and impaired ATP synthesis) (Wu and Xing, 2012; Zuo et al., 2014). Previous studies have shown that ROS generated after brief exposure to ELF-EMF play a critical role in cell proliferation, and may represent an initial cellular event (Wolf et al., 2005). In contrast, continuous ROS production resulting from long-term ELF-EMF exposure can lead to accumulation of DNA damage and slow cell-cycle progression (Huang et al., 2014). In normal cells exposed to extremely low-frequency microwave radiation, mTOR expression levels are markedly increased; canonical mTOR-regulated pathways (such as PI3K/Akt) are upregulated (Mansou et al., 2022), ERK signaling is activated, and the proportion of cells in S-phase is elevated (Patruno et al., 2015). However, whether the mTOR pathway responds similarly to high-power microwave exposure remains to be investigated.

As the only transparent tissue directly exposed to the external environment, the eye is highly sensitive to electromagnetic radiation, closely linked to its anatomical features. The corneal epithelium not only maintains corneal dehydration and refractive properties but also serves as the frontline barrier against physical stimuli such as radiation and electromagnetic interference (Yam and Kwok, 2014; Um et al., 2014; Tenkate, 1999). Existing research has shown that high-frequency millimeter waves (40–95 GHz) can induce epithelial detachment, corneal thinning, edema, and opacity in rabbit corneas (Kojima et al., 2018), while exposure at even higher frequencies (162 GHz) has been associated with fluorescein staining and corneal opacification (Kojima et al., 2020). However, these studies have mainly focused on thermal effects and have not addressed the unique protective or signaling mechanisms of the corneal layers in response to HPM (Kojima et al., 2018). Only a few studies have explored the effects of microwaves at common frequencies (e.g., 2.45 GHz) on corneal endothelial cells, such as endothelial abnormalities in monkeys (Kues et al., 1985)and slight epithelial thickening in rats (Akar et al., 2013). Overall, the impact of HPM on the corneal epithelium remains largely unexplored, highlighting the need to investigate its protective mechanisms and cellular responses.

In addition, most existing studies on high-power microwave bio-effects have concentrated on S-band (Wang et al., 2013; Qiao et al., 2014; Wang et al., 2017; Zhi et al., 2021) and X-band (Pakhomov et al., 2002; Sun et al., 2022a; Sun et al., 2022b) frequencies, whereas research specifically investigating C-band HPM remains sparse. Therefore, we systematically investigated the biological effects of 4.3 GHz HPM on HCE-T cells, focusing on cell viability, oxidative stress, mitochondrial dysfunction, apoptosis, and transcriptomic alterations, with the aim of elucidating the mechanisms of HPM-induced cellular injury.

## 2 Materials and methods

### 2.1 Materials

Dulbecco’s modified Eagle’s medium (DMEM), fetal bovine serum (FBS) and Penicillin-Streptomycin were purchased from Gibco (Thermo Fisher Scientific, Rockville, MD, USA). Cell Counting Kit-8 (CCK-8) assay kit, Reactive Oxygen Species Assay Kit, Annexin V-PE/7-AAD Apoptosis Detection Kit and JC-10 Mitochondrial Membrane Potential Assay Kit were from Yeasen Biotech Company, Shanghai, China. mTOR (1:1000, ab32028),p-mTOR (Ser2448) (1:2000, ab109268),p-AMPK (1:1000, Thr172) (ab133448) were from Abcam, Cambridge, UK. ULK1 (1:500, 20986-1-AP), p-ULK1 (Ser555) (1:500, 80218-1-RR), TSC2 (1:1000, 24601-1-AP), p-TSC2 (1:500, 29000-1-AP), AMPK (1:5000, 10929-2-AP) were from Proteintech, Wuhan, China.β-actin (1:5000, GB15003-100), HRP conjugated Goat Anti-Rabbit IgG (H + L) (1:3000, GB23303) were from Servicebio, Wuhan, China.

### 2.2 Pulsed HPM generator and experimental details

The microwave exposure system is mainly composed of a microwave source (GuoruiZhaofu Electronic, Wuhu, China), an amplifier (GuoruiZhaofu Electronic), a horn antenna, and an irradiation platform.

**FIGURE 1:**
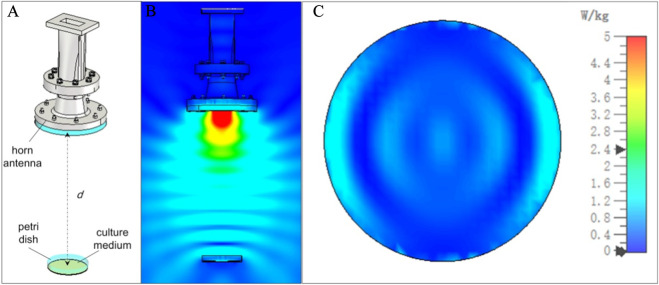
Schematic diagram of the irradiation setup **(A)** Electric field distribution in the longitudinal section when a microwave signal with an average power of 0.5 W is injected **(B)** and SAR distribution at the bottom of the culture medium when a microwave signal with an average power of 0.5 W is injected **(C)**.

An electromagnetic simulation software was used to establish the irradiation model, which mainly consists of a horn antenna, a 9 cm petri dish, and a culture medium, as shown in Figure 1. The main material of the antenna is selected as PEC (Perfect Electric Conductor). The dielectric constant of the antenna window material is 2.3, the dielectric constant of the culture medium is 70.7 with a conductivity of 5.8 S/m, and the dielectric constant of the petri dish material is 2.65. The distance (d) between the bottom of the petri dish and the antenna aperture is 33 cm, and the volume of the culture medium in the petri dish is 8 mL. The antenna radiates pulsed waves with adjustable repetition frequency and a pulse width of 100 ns. The peak microwave power density formed at the central position 33 cm away from the antenna aperture is approximately 1080 W/cm^2^. The irradiation simulation model is shown in Figure 1. For the sake of calculation simplicity, a microwave signal with a default average power of 0.5 W is injected at the antenna port, and the electric field distribution of its longitudinal section is shown in Figure 1.

Since cells grow adherently on the bottom of the culture medium, the Specific Absorption Rate (SAR) at the bottom of the culture medium was monitored during the irradiation process. Due to the extremely small size of cells relative to the microwave wavelength, it is impractical to model them. Additionally, since both cells and the culture medium are primarily composed of water, their dielectric constants and conductivities are not significantly different. Therefore, during the simulation, it was assumed that the SAR at the bottom of the culture medium corresponds to the SAR of cells at the same location.

It can be observed that the SAR distribution forms an annular pattern with stronger intensity on both sides and weaker intensity in the upper and lower regions. To ensure that cells at the same radial position receive a comparable dose during prolonged irradiation, the petri dish was maintained in uniform and slow rotation around its central axis. This irradiation method allows the SAR values at the bottom of the culture medium to form an annular distribution over an extended period (Figure 1 only shows the SAR distribution at one rotation angle, with distributions at other angles being consistent with this figure). Calculations indicate that the overall average SAR value at the bottom of the culture medium is 0.43 W/kg.

Given that the actual microwave peak power injected into the laboratory horn antenna is P = 1910000 W with pulsed waves of 100 ns pulse width (t) and adjustable repetition frequency, the average instantaneous SAR peak value of cells at the bottom of the culture medium can be calculated as (1910000/0.5) × 0.43 = 1,642,600 W/kg. The average SAR can be calculated by multiplying the instantaneous SAR peak by the pulse repetition frequency and pulse width. For repetition frequencies of 10 Hz, 20 Hz, and 50 Hz, the corresponding average SAR values are 1.64 W/kg, 3.28 W/kg, and 8.2 W/kg.

### 2.3 Cell culture

The human corneal epithelial cell-transformed (HCE-T) line was obtained from Procell Life Science & Technology Co., Ltd. (Wuhan, China). Cells were maintained in Dulbecco’s Modified Eagle Medium (DMEM, 1×; Gibco, USA) supplemented with 10% fetal bovine serum (FBS), 100 μg/mL streptomycin, and 100 U/mL penicillin. Cultures were incubated at 37 °C in a humidified atmosphere containing 5% CO_2_ and 95% air. Cells were subcultured every 2∼3 days in 90 mm culture dishes.

### 2.4 Temperature monitoring

**FIGURE 2:**
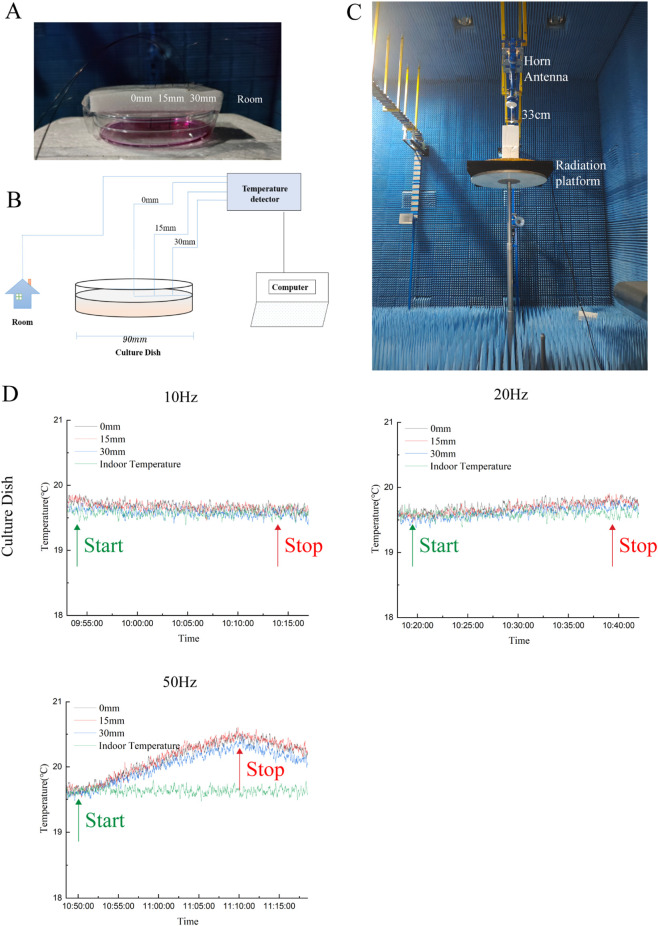
Temperature measurement in a 90-mm culture dish **(A)** Schematic diagram of temperature measurement in a 90-mm culture dish **(B)** Photograph of 4.35 GHz HPM irradiation setup **(C)** Temperature measurement results **(D)**.

Temperature changes at the bottom of the 90 mm culture dishes during HCE-T cell exposure were continuously monitored in real time using a Luxtron M3300 fiber optic temperature sensor (Lumasense Technology, Santa Clara, CA), as shown in Figures 2A–C. This device allowed precise measurement of temperature fluctuations at the culture dish bottom throughout the experiment. To ensure uniform irradiation dose, the experimental setup was designed as follows: the distance between the antenna and the culture dish was fixed at 33 cm, and three holes (diameter 0.5 mm) were drilled at radial distances of 0 mm, 15 mm, and 30 mm from the center of the dish. A miniature temperature probe was sequentially inserted into these holes to monitor real-time temperature changes on the culture medium surface during exposure. Temperature data were recorded continuously throughout the 20 min irradiation period.

### 2.5 Cell viability assay

Cell proliferation and viability were assessed using a Cell Counting Kit-8 (CCK-8; Yeasen Biotech, Shanghai, China). HCE-T cells were exposed to HPM at different repetition frequencies (control, 1.64 W/kg, 3.28 W/kg and 8.2 W/kg) and analyzed at 6, 24, and 48 h post-irradiation. Cells were seeded into 96-well plates at a density of 1 × 10^3^ cells per well, followed by the addition of 100 μL CCK-8 reagent. After incubation for 1 h at 37 °C in a humidified atmosphere containing 5% CO_2_, absorbance was measured at 450 nm using a microplate reader. Each group was tested in six replicate wells.

### 2.6 Intracellular ROS detection

Intracellular ROS levels were measured using a Reactive Oxygen Species Assay Kit (Beyotime, Shanghai, China). HCE-T cells were exposed to HPM at different repetition frequencies (control, 1.64 W/kg, 3.28 W/kg and 8.2 W/kg) and analyzed at 6, 24, and 48 h post-irradiation. Cells were seeded into 96-well plates at a density of 1 × 10^3^ cells per well, and incubated with DCFH-DA working solution according to the manufacturer’s instructions. After incubation for 1 h at 37 °C in a humidified atmosphere containing 5% CO_2_, fluorescence intensity was measured at an excitation wavelength of 488 nm and an emission wavelength of 525 nm using a microplate reader. Each group was tested in six replicate wells.

### 2.7 Cell apoptosis assay

Cell apoptosis was evaluated using an Annexin V-PE/7-AAD Apoptosis Detection Kit (BD Biosciences, San Jose, CA, USA). This assay is based on fluorescence detection of phosphatidylserine exposure, which serves as an indicator of apoptosis. Briefly, HCE-T cells (1.5 × 10^6^) were seeded into 90 mm culture dishes and incubated for 24 h at 37 °C in a humidified atmosphere containing 5% CO_2_. Cells were then exposed to HPM at different repetition frequencies (control, 1.64 W/kg、3.28 W/kg和8.2 W/kg), with three replicate dishes per group. At 6 h and 24 h post-irradiation, cells were harvested using EDTA-free trypsin and stained with Annexin V-PE and 7-AAD according to the manufacturer’s instructions. Apoptotic cells were analyzed using flow cytometry at an excitation wavelength of 546 nm and an emission wavelength of 647 nm (FL3 channel).

### 2.8 Mitochondrial membrane potential staining

Mitochondrial membrane potential (MMP) was assessed using a JC-10 Mitochondrial Membrane Potential Assay Kit (Beyotime, Shanghai, China) to evaluate early apoptotic events. HCE-T cells (1.5 × 10^6^) were seeded into 90 mm culture dishes and incubated for 24 h at 37 °C in a humidified atmosphere containing 5% CO_2_. Cells were then exposed to HPM at different repetition frequencies (control, 1.64 W/kg、3.28 W/kg和8.2 W/kg), with six replicate dishes per group. At 6 h and 24 h post-irradiation, cells were stained with the JC-10 probe according to the manufacturer’s instructions. Fluorescence signals were detected using a microplate reader: red JC-10 aggregates in normal mitochondria (excitation 540 nm, emission 590 nm) and green JC-10 monomers in depolarized mitochondria (excitation 490 nm, emission 525 nm). The ratio of red to green fluorescence intensity was used to quantify mitochondrial damage. In addition, live-cell imaging was performed to visualize and calculate fluorescence intensity.

### 2.9 Transcriptome sequencing

After quality assessment of the RNA samples, total RNA was extracted and mRNA was enriched using Oligo(dT) magnetic beads. The enriched mRNA was randomly fragmented with divalent cations in Fragmentation Buffer, and the resulting fragments were used as templates to synthesize cDNA libraries following the standard NEB library preparation protocol. The constructed libraries were first quantified and diluted to 1.5 ng/μL, and the insert size was assessed. Quantitative real-time PCR (qRT-PCR) was then used to determine the effective concentration of each library, ensuring that the concentration exceeded 2 nM. Qualified libraries were sequenced based on the sequencing-by-synthesis (SBS) principle, in which fluorescently labeled dNTPs, DNA polymerase, and adaptor primers were incorporated to generate amplified products, and the sequencing instrument recorded the emitted fluorescence signals to determine nucleotide sequences. Library preparation and sequencing were performed by Novogene (Beijing, China) and OE Biotech (Shanghai, China).

### 2.10 Quantification of mRNAs by real-time PCR

Total RNA was extracted using RNAzol reagent (Omega, USA), and cDNA was synthesized using a cDNA synthesis kit (TOYOBO, Japan). Quantitative real-time PCR (RT-qPCR) was performed on a CFX Opus 96 Real-Time PCR System (BIO-RAD, USA) using SuperReal PreMix Plus-SYBR Green (TIANGEN Biotech Co., Ltd., Beijing, China), following the manufacturer’s instructions. Actin was used as the internal control, and relative gene expression levels were calculated using the 2^^−ΔΔCt^ method. All experiments were conducted in triplicate. Primers used in this study are listed in Table 1.

**TABLE 1:** Sequences of primers used for quantitative real-time PCR analysis.

TSC1 F	GAC​GCC​TCC​TCC​TGC​CTC​TG
TSC1 R	GCC​TGC​CTG​CCT​CTG​GTT​TG
TSC2 F	TGT​CCG​AAC​GAG​GTG​GTG​TCC
TSC2 R	AGG​TCT​GGA​GCT​GCT​GAA​GGA​G
RHEB F	GTG​AAG​ATG​TGG​CCA​CAG​GA
RHEB R	AGG​GCC​ACT​CAG​CTT​CAA​TC
RPTOR F	CGT​AGC​CGA​CAA​GGA​CAG​CAT​C
RPTOR R	CGT​CAG​CAG​AAG​CGA​GCA​GTC
PPARG F	GCC​CAG​GTT​TGC​TGA​ATG​TG
PPARG R	TGA​GGA​CTC​AGG​GTG​GTT​CA
ULK1 F	CGC​CAA​CCC​CAA​CAG​CAT​CC
ULK1 R	TCC​GCC​TTC​CCG​TCG​TAG​TG

### 2.11 Western blot

Total proteins from HCE-T cells were extracted using RIPA lysis buffer supplemented with protease and phosphatase inhibitors. Protein concentrations were measured using the BCA assay and normalized to equal amounts. Samples were then mixed with 5× loading buffer, boiled at 95 °C for 5 min, separated by SDS–PAGE, and transferred onto PVDF membranes.

Membranes were blocked with 5% skim milk (for total-protein antibodies) or 5% BSA (for phospho-specific antibodies) at room temperature for 2 h, and then incubated overnight at 4 °C with the following primary antibodies: mTOR, p-mTOR, AMPK, p-AMPK, ULK1, p-ULK1, TSC2, p-TSC2. After washing, membranes were incubated with HRP-conjugated secondary antibodies for 1 h at room temperature. Protein bands were visualized using an ECL chemiluminescence kit with a FluorChem R imaging system (ProteinSimple, USA). Band intensities were quantified using ImageJ 1.54p (NIH, USA).

### 2.12 Statistical analysis

All experiments were independently repeated at least three times, and data are presented as mean ± standard deviation (Mean ± SD). For cell viability and ROS assays, values were normalized to the corresponding control group at each time point.

Statistical differences among groups were evaluated using one-way analysis of variance (ANOVA), followed by Tukey’s multiple comparisons test when the ANOVA indicated significance. A value of P < 0.05 was considered statistically significant. All analyses were performed using Graph Pad Prism.

## 3 Results

### 3.1 Temperature changes during HPM exposure

As shown in Figure 2D, under 1.64 W/kg and 3.28 W/kg irradiation, the real-time temperature in 90 mm culture dishes remained stable at approximately 19.0∼20.0 °C, consistent with ambient room temperature fluctuations, with no noticeable increase. Under 8.2 W/kg irradiation, the temperature gradually increased, reaching a peak of 20.8∼21.0 °C after approximately 20 min of exposure (an increase of ∼0.8 °C–1.0 °C relative to the initial value), and gradually returned to baseline after the irradiation ceased. In summary, except for the minor and transient temperature rise observed under 8.2° W/kg exposure in the specific dish setup (<1 °C), the cellular responses under these experimental conditions were generally not driven by thermal effects, but primarily reflected non-thermal biological effects of microwave exposure.

### 3.2 HPM exposure reduces HCE-T cell viability

**FIGURE 3:**
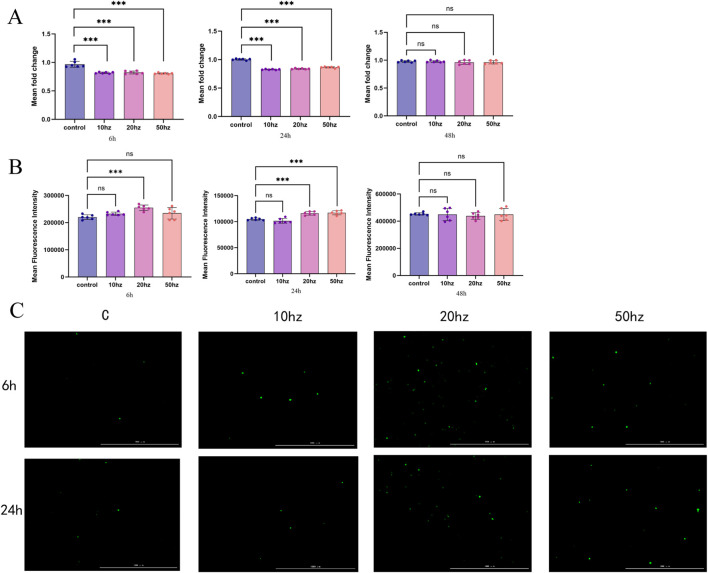
Changes in cell viability of HCE-T cells at 6 h, 24 h, and 48 h after 20-min exposure to 4.35 GHz HPM at different pulse repetition frequencies (10 Hz, 20 Hz, 50 Hz) **(A)** Changes in intracellular ROS levels of HCE-T cells at 6 h, 24 h, and 48 h after 20-min exposure to 4.35 GHz HPM at different pulse repetition frequencies (10 Hz, 20 Hz, 50 Hz) **(B)** Representative fluorescence images of ROS in HCE-T cells at 6°h and 24 h after 20-min exposure to 4.35 GHz HPM at different pulse repetition frequencies (10 Hz, 20 Hz, 50 Hz) **(C)**. Data are presented as mean ± SD (n = 6). *p < 0.05, **p < 0.01, ***p < 0.001 (one-way ANOVA with Tukey’s multiple comparisons test).

As shown in Figure 3A, 6 h after HPM exposure, cell viability in all treatment groups was significantly lower than that in the control group (*p <* 0.001). Among them, the 8.2 W/kg group exhibited the most pronounced inhibitory effect, with cell viability decreasing to approximately 80.1% of the control. At 24 h post-irradiation, cell viability remained significantly reduced in all treatment groups, although the degree of inhibition slightly alleviated; the 8.2 W/kg group recovered to about 86.5% of control levels.

At 48 h post-irradiation, cells generally showed a trend of recovery. Notably, the 3.28 W/kg group displayed the most sustained and significant proliferation inhibition, with cell viability at approximately 93.2%, significantly lower than both the control group (*p <* 0.01) and other frequency groups. In contrast, cell viability in the 1.64 W/kg and 8.2 W/kg groups had nearly returned to normal levels at 48 h, showing no significant difference compared with the control group.

### 3.3 HPM exposure induces intracellular ROS generation in HCE-T cells

As shown in Figures 3B,C, 6 h after HPM exposure, intracellular ROS levels were significantly elevated in all treatment groups (*p <* 0.01). The 3.28 W/kg group exhibited the highest ROS levels, reaching 1.22-fold of the control. The 8.2 W/kg group also showed a strong induction effect, with ROS levels at 1.18-fold of the control, while the 1.64 W/kg group showed a relatively weaker effect (1.10-fold of the control).

At 24 h post-irradiation, ROS levels displayed differential changes among the groups. ROS levels in the 3.28 W/kg and 8.2 W/kg groups remained significantly higher than the control (1.14- and 1.15-fold, respectively, p < 0.05), whereas ROS levels in the 1.64 W/kg group had returned to a level not significantly different from the control. By 48 h post-irradiation, the ROS levels showed a clear shift: the 1.64 W/kg and 3.28 W/kg groups dropped below control levels (0.95- and 0.94-fold, respectively), whereas the 8.2 W/kg group exhibited no significant difference compared with the control. These patterns suggest that microwaves at different frequencies may affect long-term cellular redox homeostasis through distinct mechanisms.

### 3.4 HPM-induced mitochondrial dysfunction and apoptosis in HCE-T cells

**FIGURE 4:**
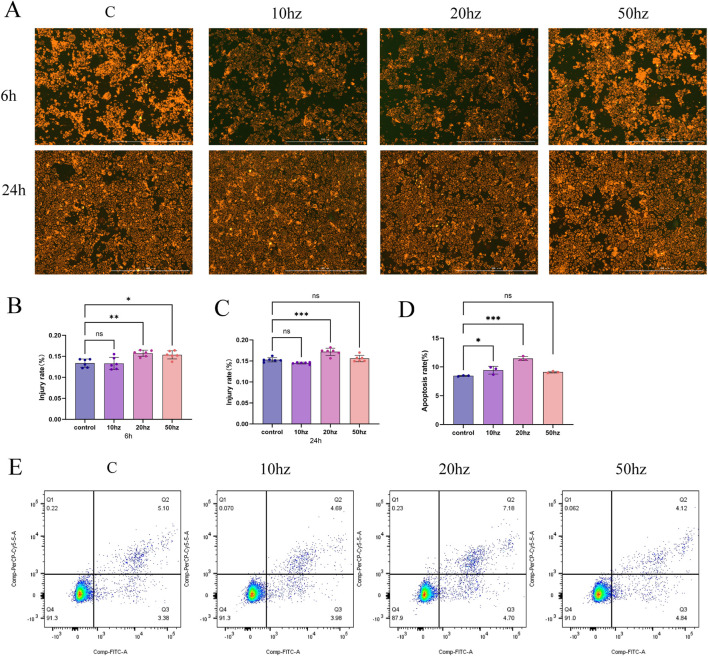
Representative JC-10 fluorescence images showing mitochondrial membrane potential (MMP) in HCE-T cells at 6 h and 24 h after a 20-min exposure to 4.35 GHz HPM (at the indicated pulse repetition frequencies) **(A)** Quantification of MMP loss at 6 h post-exposure, expressed as the red/green fluorescence ratio **(B)** Quantification of MMP loss at 24 h post-exposure, expressed as the red/green fluorescence ratio **(C)** Quantification of apoptosis rates in HCE-T cells at 24 h post-exposure determined by Annexin V-PE/7-AAD flow cytometry **(D)** Representative Annexin V-PE/7-AAD apoptosis staining images at 24 h post-exposure **(E)**. Data are presented as mean ± SD; the number of independent experiments and the statistical tests used are indicated in the figure. (n = 3, *p < 0.05, **p < 0.01, ***p < 0.001)

As shown in Figures 4A–C, JC-10 staining revealed significant changes in mitochondrial membrane potential (MMP) as early as 6 h after HPM exposure. The 3.28 W/kg group exhibited the most pronounced decrease in the red/green fluorescence ratio, indicating the most severe loss of MMP. At 24 h post-irradiation, the 3.28 W/kg group maintained the lowest MMP, with a significant difference compared to the control (p < 0.001).

Notably, the degree of MMP loss was highly consistent with the subsequently observed apoptosis rates. The 3.28 W/kg group exhibited the most severe mitochondrial dysfunction at the early stage (6 h), which corresponded well with the highest apoptosis rate observed at 24 h.

As shown in Figures 4D,E, at 24 h post-irradiation, apoptosis rates differed significantly among the groups. The 3.28 W/kg group displayed a significantly higher apoptosis rate than all other groups (p < 0.01), reaching 11.47%, which represents an approximately 35.7% increase compared with the control group (8.45%). Although the 1.64 W/kg and 8.2 W/kg groups showed slight increases in apoptosis (9.43% and 9.10%, respectively), these differences were not statistically significant compared with the control.

### 3.5 Transcriptomic analysis reveals frequency-dependent molecular responses to HPM

**FIGURE 5:**
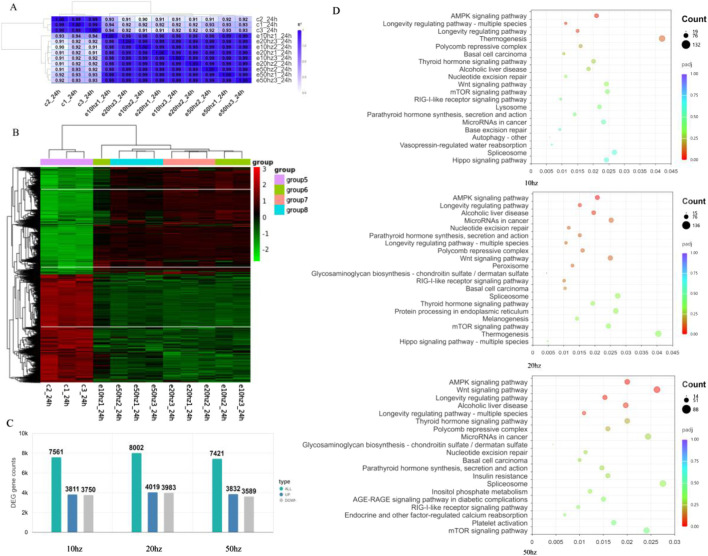
Sample correlation analysis showing a heatmap of pairwise correlations among biological replicates **(A)** Hierarchical clustering of differentially expressed genes (DEGs), presented as a heatmap to illustrate expression patterns across treatment groups **(B)** Differential gene expression analysis results, presented as a bar chart summarizing the numbers of upregulated and downregulated genes across the exposure conditions **(C)** KEGG pathway enrichment analysis showing the top significantly enriched pathways **(D)**.

To elucidate the molecular mechanisms underlying HPM-induced effects on cellular function, transcriptome sequencing and KEGG pathway enrichment analysis were performed to systematically compare signaling pathway alterations in HCE-T cells exposed to different pulse repetition frequencies (1.64 W/kg, 3.28 W/kg, and 8.2 W/kg). Sample correlations and gene clustering results are shown in Figures 5A,B, while the number of differentially expressed genes (DEGs) is presented in Figure 5C. At a fold-change threshold of 0.58, the 1.64 W/kg group exhibited 3,832 upregulated genes and 3,589 downregulated genes; the 3.28 W/kg group showed 4,019 upregulated and 3,983 downregulated genes; and the 8.2 W/kg group displayed 3,811 upregulated and 3,750 downregulated genes.

As shown in Figure 5D, HPM exposure broadly affected cellular signaling networks, with pronounced frequency-specific effects. In the 1.64 W/kg group, the most significantly enriched pathways included AMPK signaling (Count = 19), longevity regulating pathway (Count = 132), and thermogenesis, while base excision repair was also notably activated. Transcriptomic analysis indicates that C-band microwave exposure first perturbs energy metabolism and activates AMPK, forming a common stress baseline. On this foundation, different repetition frequencies induce distinct cellular fates: 1.64 W/kg primarily promotes repair and adaptation, maintaining cellular homeostasis; 3.28 W/kg induces extensive DNA and protein damage, accompanied by Polycomb-mediated chromatin remodeling, thereby triggering irreversible cell cycle arrest and senescence, which explains the sustained proliferation inhibition observed at 24 h; 8.2 W/kg mainly disrupts metabolic signaling and cellular functions (adhesion, cytoskeleton, calcium), resulting in early but partially reversible functional impairments.

Transcriptomic analysis revealed that the AMPK signaling pathway was significantly enriched across all exposure groups (1.64 W/kg, 3.28 W/kg, and 8.2 W/kg) compared with the control, suggesting a common stress response to HPM irradiation. In the 1.64 W/kg group, AMPK activation was accompanied by enrichment of DNA repair pathways (e.g., nucleotide and base excision repair) and longevity-regulating pathways, indicating that AMPK primarily functioned in promoting adaptive repair and maintaining genomic stability under relatively mild stress. In contrast, the 3.28 W/kg group exhibited concurrent enrichment of the mTOR signaling pathway and Polycomb repressive complex, together with strong suppression of mTOR-related genes, suggesting that AMPK activation here was linked to mTOR inhibition, induction of autophagy, and irreversible cell cycle arrest, consistent with the sustained reduction in cell proliferation observed at 24 h. In the 8.2 W/kg group, AMPK enrichment was associated with pathways related to metabolic regulation, including insulin resistance and Wnt signaling, implying that AMPK acted mainly to reprogram energy metabolism and facilitate recovery after acute oxidative stress. These results indicate that although AMPK signaling represents a common target of HPM exposure, its downstream effects vary with repetition frequency, driving adaptive repair at 1.64 W/kg, stress-induced autophagy and senescence at 20 Hz, and metabolic compensation at 8.2 W/kg.

This frequency-dependent response spectrum suggests that distinct microwave parameters differentially interfere with energy, genomic, and proteostasis pathways, ultimately determining cell fate.

### 3.6 Frequency-dependent alterations in key mTOR and metabolic pathway genes

**FIGURE 6:**
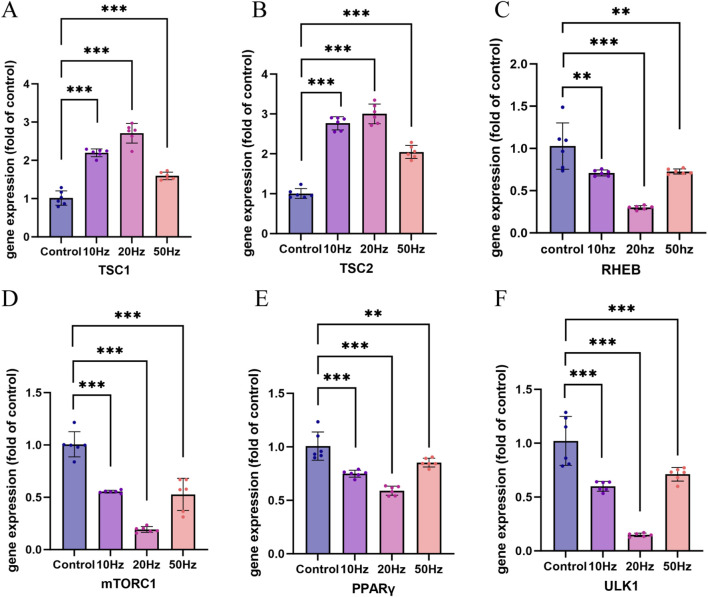
qRT-PCR validation of mTOR pathway-related gene expression. **(A)** TSC1; **(B)** TSC2; **(C)** RHEB; **(D)** mTOR; **(E)** PPARγ; **(F)** ULK1. (n = 6, *p < 0.05, **p < 0.01, ***p < 0.001)

Given that AMPK signaling emerged as the most significantly altered pathway across all three exposure levels, we prioritized this axis for further investigation. Because AMPK functions as a central energy sensor and upstream regulator of the TSC1/TSC2 complex and mTOR signaling. We focused our subsequent validation efforts on TSC1 and TSC2, and downstream components of the mTOR axis. Based on the results of the three exposure conditions, the present study focused on a detailed validation of the mTOR signaling pathway. As shown in Figure 6, compared with the control group, HPM exposure significantly altered the mRNA levels of TSC1 and TSC2 (p < 0.05). The 3.28 W/kg group exhibited the most pronounced effect: TSC1 expression was upregulated by approximately 2.7-fold, and TSC2 expression increased by approximately 3.0-fold, indicating that this frequency most strongly activated the upstream signals that inhibit the mTOR pathway.

### 3.7 Protein-level confirmation of AMPK–TSC2–mTOR pathway suppression at 24 h

**FIGURE 7:**
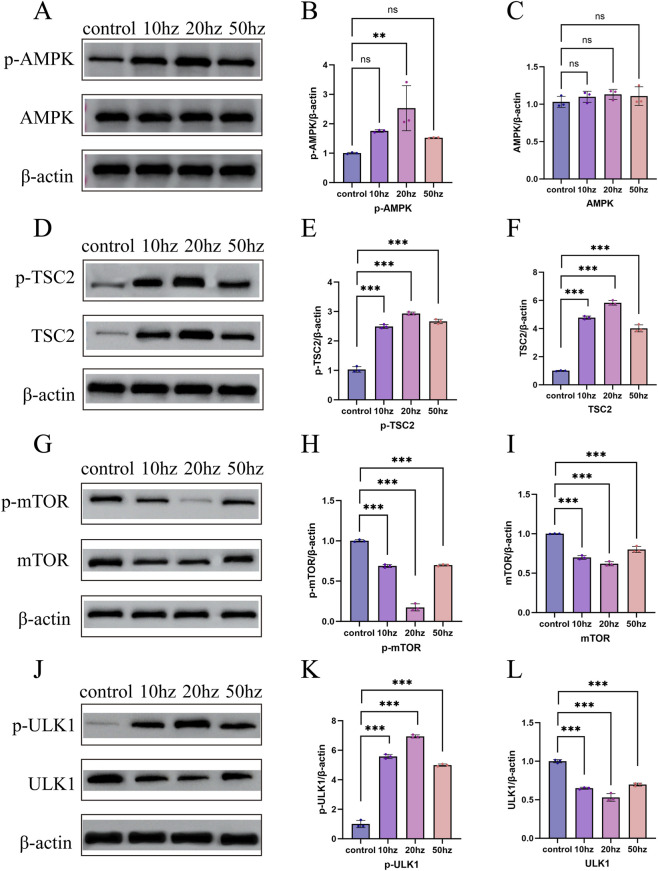
Protein-level validation of the AMPK–TSC2–mTOR–ULK1 signaling pathway at 24 h after 4.3 GHz HPM exposure. Representative Western blot bands and quantification of total and phosphorylated signaling proteins, including AMPK, phosphorylated AMPK (p-AMPK) **(A–C)** TSC2, and phosphorylated TSC2 (p-TSC2) **(D–F)** mTOR, phosphorylated mTOR (p-mTOR) **(G–I)** ULK1, phosphorylated ULK1 (p-ULK1) **(J–L)** in HCE-T cells following exposure to 1.64 W/kg, 3.28 W/kg, and 8.2 W/kg HPM for 24 h. Protein expression levels were normalized to the corresponding total protein (for phosphorylated forms) or β-actin (for total protein). (n = 3, *p < 0.05, **p < 0.01, ***p < 0.001).

Western blot analysis further validated the key protein alterations in the AMPK–TSC2–mTOR–ULK1 signaling pathway in Figure 7. Total AMPK levels showed no substantial differences among treatment groups; however, its phosphorylated form (p-AMPK) increased in a dose-dependent manner (Figures 7A–C). All exposure groups (1.64 W/kg, 3.28 W/kg, and 8.2 W/kg) exhibited higher p-AMPK/β-actin ratios than the control, with the 3.28 W/kg group showing the most prominent elevation. Similarly, both TSC2 and phosphorylated TSC2 (p-TSC2) were markedly upregulated in all exposure groups, again with the highest levels observed in the 3.28 W/kg group (Figures 7D–F).

In contrast, mTOR and p-mTOR expression decreased progressively across the three doses (Figures 7G–I). The 3.28 W/kg group displayed the strongest inhibition, with p-mTOR reduced to the control level, indicating pronounced suppression of mTORC1 activity. ULK1 expression showed a similar pattern: total ULK1 decreased in the 1.64 W/kg and 3.28 W/kg groups, whereas phosphorylated ULK, the activated form (Figures 7J–L), was markedly upregulated in the 3.28 W/kg group, further supporting AMPK-mediated autophagy initiation.

Overall, the Western blot findings are consistent with the transcriptomic and qPCR data: HPM exposure activates the AMPK pathway, enhances upstream inhibitory signals (TSC2/p-TSC2), suppresses mTORC1 activity, and promotes autophagy activation through ULK1 phosphorylation, with the 3.28 W/kg dose inducing the most pronounced signaling changes.

## 4 Discussion

The eye is a vital organ of the human body, an extraordinarily precise and complex sensory apparatus, whose core function is to receive light signals and convert them into neural signals interpretable by the brain, thereby generating vision (Yang et al., 2018; Schwend, 2023). The cornea constitutes the transparent front one-sixth of the eyeball, acting like a convex glass window. It is avascular, yet it is richly innervated, making it extremely sensitive to external stimuli (Guerrero-Moreno et al., 2020; Cursiefen et al., 2006). Many studies to date have reported that high-power microwave (HPM) exposure can cause damage to neural cells, including hippocampal neurons, neural stem cells, and glial cells in the central and peripheral nervous system (Gao et al., 2025). These injuries are mainly associated with oxidative stress, mitochondrial dysfunction, apoptosis signaling pathways (such as intrinsic apoptosis involving Caspase-3/Bax/Bcl-2 (Rana et al., 2023), DNA damage and repair routes [e.g., ATM/ATR and p53-regulated pathways (Rana et al., 2024)], and inflammatory responses. Based on these reasons, this study aims to investigate the damaging effects of HPM on corneal epithelial cells. Our findings demonstrate that among tested pulse repetition frequencies, 3.28 W/kg HPM exposure causes the most severe non-thermal damage in human corneal epithelial cells, characterized by early oxidative stress, mitochondrial dysfunction, elevated apoptosis, and lasting proliferation inhibition.

HPM exposure can induce diverse biological effects depending on the power density and pulse repetition frequency. In this study, we observed a nonlinear dose–response pattern, where the 3.28 W/kg group caused more severe cellular damage than either the 1.64 W/kg group or the 8.2 W/kg group exposures. This paradoxical response suggests that biological injury from HPM does not necessarily increase with power intensity but may instead involve resonant-type interactions between the microwave field and subcellular structures. The pronounced effects observed under moderate-power exposure likely arise from highly efficient energy coupling. One hour after irradiation, the 3.28 W/kg group exhibited the strongest ROS burst, which directly attacked mitochondria, resulting in marked loss of mitochondrial membrane potential (ΔΨm) (Costa et al., 2024) and triggering intrinsic apoptosis. Consistently, mitochondrial dysfunction was most severe at 6 h and 24 h post-exposure, coinciding with the highest apoptosis rate (Dho et al., 2025; Li et al., 2024). Excessive ROS likely originated from perturbations in plasma membrane ion channels and the mitochondrial electron transport chain, amplifying oxidative stress.

We hypothesize that this may result from a resonance-type interaction between the 4.3 GHz pulsed field and cellular biomolecular oscillators, such as voltage-gated calcium channels (VGCCs) (Bertagna et al., 2021) or mitochondrial respiratory complexes (Wood and Karipidis, 2021). This coupling may transiently synchronize oscillations, enhancing Ca^2+^ influx and electron leakage, which leads to intracellular calcium overload, ΔΨm collapse, and activation of caspase-dependent apoptosis (Pall, 2013). Severe mitochondrial damage thus drives irreversible intrinsic apoptosis, explaining the maximal cell death at 24 h (Costa et al., 2024).

From a long-term perspective, the extensive DNA and organelle damage observed after 3.28 W/kg exposure is consistent with the enrichment of longevity-associated and Polycomb complex pathways and the sustained inhibition of cell proliferation at 24 h (CCK-8 results). However, because we did not directly measure canonical senescence markers (e.g., p16^INK4a, SA-β-galactosidase activity), the suggestion that these molecular changes reflect activation of irreversible cell cycle arrest or a full senescence program remains hypothesis-generating rather than definitive. Therefore, this interpretation should be considered a potential mechanism supported by pathway analysis and proliferation data, and requires further experimental validation. Transcriptomic analysis also revealed strong suppression of the mTOR pathway, with upregulation of TSC2 and downregulation of mTOR, suggesting activation of the AMPK–TSC2–mTOR axis as an energy-conserving “brake” under extreme oxidative stress. ROS can rapidly inhibit mTORC1 via the ATM–LKB1–AMPK–TSC2 cascade, promoting autophagy (Alexander et al., 2010; Alexander et al., 2009). The Western blot findings further support the mechanism predicted from the transcriptomic analysis: all exposure doses activated the AMPK pathway and increased TSC2 expression, thereby inhibiting mTORC1 signaling. Among them, the 3.28 W/kg dose produced the most coherent and robust cascade response, characterized by strong p-AMPK and p-TSC2 induction, accompanied by a dramatic reduction in p-mTOR. This suggests that cells exposed to this moderate dose activate the classical AMPK–TSC2–mTOR energy-sensing axis as a compensatory response to oxidative and metabolic stress.

In contrast, the 1.64 W/kg group exposure induced only mild oxidative stress and mitochondrial dysfunction, and activated DNA repair pathways such as base excision repair (BER), allowing cells to maintain genomic integrity and survival without significant apoptosis (Shen et al., 2016). The 8.2 W/kg group exposure primarily perturbed metabolic and membrane receptor signaling rather than causing severe mitochondrial or DNA damage (Huyan et al., 2020; Sendera et al., 2024). ROS levels in this group returned to control levels by 48 h, suggesting that repair and metabolic adaptation restored homeostasis (Alexander et al., 2010; Kleiber, 2017). Therefore, 8.2 W/kg effects likely represent early acute stress followed by late compensation, whereas 3.28 W/kg exposure maximizes intracellular stress via resonance-like amplification, resulting in the highest oxidative and apoptotic injury (Elexpuru-Zabaleta et al., 2023). As HCE-T cells are immortalized and not fully equivalent to primary corneal epithelial cells, this limitation should be considered when interpreting the biological relevance of our findings.

## 5 Conclusion

In summary, our study demonstrates that pulse repetition frequency (PRF) is a critical determinant of the biological effects of high-power microwaves (HPM) on human corneal epithelial cells (HCE-T). Among the tested frequencies, 3.28 W/kg induced the most severe and persistent cellular damage, characterized by early intense oxidative stress, pronounced mitochondrial dysfunction, the highest apoptosis rate, and prolonged proliferation inhibition. These findings highlight the necessity of considering PRF, in addition to frequency and power, when assessing the biological risks of HPM exposure.

## 6 Future directions

Further studies are warranted to elucidate the underlying molecular mechanisms at the protein level, particularly the phosphorylation dynamics of AMPK and ULK1, and to validate the involvement of autophagy pathways. Investigating the effects of different PRFs on other ocular cell types or *in vivo* models will provide a more comprehensive understanding of HPM-induced ocular hazards. Moreover, these insights could inform the development of safer exposure guidelines and potential protective strategies against HPM-induced cellular damage.

## Data Availability

The original contributions presented in the study are included in the article/Supplementary Material, further inquiries can be directed to the corresponding author. The raw sequence data reported in this paper have been deposited in the Genome Sequence Archive (Genomics, Proteomics & Bioinformatics 2025) in National Genomics Data Center (Nucleic Acids Res 2025), China National Center for Bioinformation / Beijing Institute of Genomics, Chinese Academy of Sciences (GSA: CRA035652) that are publicly accessible at https://ngdc.cncb.ac.cn/gsa or https://ngdc.cncb.ac.cn/gsa/browse/CRA035652 (Zhang et al., 2025; CNCB-NGDC Members and Partners, 2024).
